# The Story of the Insane from Year to Year

**Published:** 1898-02-12

**Authors:** 


### THE STORY OF THE INSANE FROM YEAR TO YEAR.

Tenth Series.

### NORTHUMBERLAND COUNTY ASYLUM AT MORPETH.

The numbers increased from 583 to 590 during the year. There were 150 first admissions, 23 readmissions, 76 recoveries, and 62 deaths. The recoveries stand at 50·6 per cent. on the admissions, which is very high for a county asylum; but the readmissions are also high. The deaths are 10·4 per cent. on the average number resident, and 23 of them were due to cerebral and spinal diseases, 16 to consumption, and 1 to suicide. Of the cases admitted, 15 owed their insanity to domestic trouble, religion, love, &c., and 36 to intemperance in drink; while hereditary tendency is the assigned cause in 27. About two years since a new system of ventilation was adopted. The mortality from consumption has not diminished yet; but then, as Dr. McDowall points out, the trial is not yet sufficiently extensive to enable one to arrive at any certain conclusions. It is evident from the general tone of the report that Dr. McDowall has lost none of his energy, and he gives a list of various alterations and improvements which he thinks the asylum will require in the near future, the most important of which are a detached hospital, better means for the extinction of fire, and additional accommodation for male patients. Fortunately, the asylum is still small enough to permit of considerable additions, not only without harm, but with positive advantage. Nursing lectures are given by the medical officers, and four nurses obtained certificates from the Medico-Psychological Association. The Visiting Committee say that "Dr. McDowall continues to carry out his onerous and responsible duties as ably as heretofore." The weekly rate of maintenance is 9s. 7½d. per head.

### CAMBRIDGE COUNTY ASYLUM.

Here the numbers were almost stationary during 1896, being 530 at the beginning of the year and 535 at the close of it. There were 89 first admissions, 15 readmissions, 49 recoveries, 8 discharged relieved, and 46 deaths. The recoveries stand at the very creditable percentage of 49·4 on the admissions, and the deaths at 8·7 per cent. on the average number resident. Of the deaths 13 were caused by cerebral and spinal diseases, 8 by consumption, 1 by pneumonia, and 1 by enteritis. Of the year's admissions the insanity was stated to be due to various moral causes in 24 instances, to intemperance in drink in 8, and to hereditary influence in 25. Dr. Rogers' report is a very short one, but we learn from it that the asylum contains more patients than it can properly accommodate, and it is clear that either additional wards must be built or a batch of patients boarded out at some other asylum. The latter plan can only be a temporary relief, and it is a very expensive one. The weekly cost of maintenance at Cambridge is 8s. 4½d. per head per week. The cost of those boarded out will be 14s.

If only twenty patients are boarded out there will be a loss to the Cambridge ratepayers of nearly £300 a year. If travelling expenses be added it will exceed that sum—and the cost of the additional buildings has only been postponed for a few years.

### THE ARGYLL AND BUTE ASYLUM AT LOCHGILPHEAD.

On January 1st, 1896, this asylum contained 412 patients, and this number only increased by two during the year. There were 48 first admissions, 10 readmissions, 18 recoveries, and 24 deaths. The recoveries stand at 30 per cent. of the admissions, and the deaths at 5 per cent. on the average number resident. Only 5 of the deaths were caused by cerebral diseases, and 6 by consumption. Turning to Table IX. we find that only 1 of the year's admissions was owing to intemperance in drink and 8 to hereditary tendency. Dr. Cameron seems to have had a very quiet year. There was no suicide and no fatal accident, indeed, only one serious accident is noted—namely, the fracture of a fore-arm. Dr. Cameron deplores the frequent changes among the nurses, and he thinks it is a pity that no satisfactory means of rendering asylum service more attractive has yet been devised. This is true enough; but it is not always the service that is at fault, but quite as often that absurd love of change which seems to be inherent in the female breast, and even pleasant surroundings, good wages, and humane treatment will not eradicate it wholly. The cost per head per week was 8s. 6¾d.

